# Supplementation of different fat sources affects growth performance and carcass composition of finishing pigs

**DOI:** 10.1186/s40104-018-0274-9

**Published:** 2018-08-21

**Authors:** Yanhong Liu, Dong Yong Kil, Victor G. Perez-Mendoza, Minho Song, James E. Pettigrew

**Affiliations:** 1 Department of Animal Science, University of California, Davis, CA USA; 2 Department of Animal Sciences, University of Illinois, Urbana, IL USA; 3 Department of Animal Nutrition and Physiology, Chung-Ang University, Anseong, South Korea; 4 Pancosma, Quincy, IL USA; 5 Department of Animal Science and Biotechnology, Chungnam National University, Daejeon, South Korea

**Keywords:** Carcass composition, Dietary fats, Energy values, Finishing pigs, Growth performance

## Abstract

**Background:**

There are various fat sources with different energy values and fatty acid compositions that may affect growth performance and carcass composition of grow-finishing pigs. A higher net energy was recently reported in choice white grease compared with soybean oil. Therefore, two experiments were conducted to determine whether practical responses confirm that difference between choice white grease and soybean oil, and to extend the observations to other fat sources.

**Results:**

In Exp. 1, pigs fed fats had lower (*P* < 0.05) average daily feed intake in phase II and overall period, greater (*P* < 0.05) gain:feed in phase I, phase II, and overall period than pigs fed the control diet. Pigs fed fats tended (*P* = 0.057) to have thicker backfat depth at the last rib than those fed control. Pigs fed 6% fats had greater (*P* < 0.01) gain:feed in phase II and overall period than pigs fed 3% fats. During phase I, pigs fed choice white grease grew faster (*P* < 0.05) than pigs fed soybean oil. In Exp. 2, pigs fed dietary fats (soybean oil, choice white grease, animal-vegetable blend, palm oil, or tallow) had greater (*P* < 0.01) gain:feed in each phase and overall period, greater (*P* < 0.01) average daily gain in phase I, but lower (*P* < 0.01) average daily feed intake in phase II an overall than pigs fed the control diets. The choice white grease also increased (*P* < 0.05) average daily gain during phase I compared with soybean oil. Pigs fed palm oil had thicker (*P* < 0.05) backfat depth at the 10^th^ rib than those fed soybean oil, animal-vegetable blend, or tallow.

**Conclusions:**

Inclusion of 6% dietary fat improved feed efficiency of finishing pigs, while different fats produced different practical results that may be consistent with their different energy values. Results from the early stage indicate that dietary fats with relatively more saturated fatty acids may provide greater energy than those with relatively more unsaturated fatty acids for growing pigs.

## Background

Supplementing dietary fats to swine diets is a practical method to improve growth rate and feed efficiency. Adding 5% or 10% fat to diets for grow-finishing pigs has been shown to increase feed efficiency but, in some cases to reduce carcass leanness [1, 2]. There are various fat sources available for swine producers to use, containing extremely diverse chemical compositions, which influence their digestibility and energy value [3–5].

Fats from animal sources are normally considered to have lower digestibility and hence lower energy value than fats from vegetable origin. The lower digestibility appears due to more saturated fatty acids in animal fats, which have lower ileal digestibility than unstaturated fatty acids [5, 6]. However, Kil et al. [7] reported higher swine net energy in choice white grease (CWG), mainly consisting of rendered pork fat, than in soybean oil (SBO). In addition, different fatty acid composition of different fat sources may impact carcass composition of growing and finishing pigs [8–10].

Therefore, the first objective of these experiments was to determine whether practical responses confirm the higher swine net energy in CWG than SBO and whether this observation extended to other fat sources. The second objective was to investigate the effects of different dietary fat sources on carcass compositions of finishing pigs.

## Methods

### Animals, housing, and experimental design

The protocols for these studies were reviewed and approved by the Institutional Animal Care and Use Committee of the University of Illinois at Urbana-Champaign. These studies were conducted in the Swine Research Center at the University of Illinois.

A total of 279 finishing barrows used in these two experiments were terminal offspring of PIC L337 boars × C22 sows (Pig Improvement Company, Hendersonville, TN). The average initial weights of pigs were 64.8 ± 6.20 kg and 73.0 ± 3.98 kg for Exp. 1 and 2, respectively. In Exp. 1, 135 pigs were randomly assigned to 5 different dietary treatments (9 pens per treatment and 3 pigs per pen). In Exp. 2, 144 pigs were randomly assigned to 6 dietary treatments (8 pens per treatment and 3 pigs per pen). Pigs had ad libitum access to feed and water. Pigs were placed in pens (2.6 m × 1.83 m in size) with a partial-slat concrete floor and equipped with a feeder and 2 nipple waterers. The experimental periods of Exp. 1 and 2 were 21 d and 19 d for phase I and 28 d and 28 d for phase II, respectively.

### Dietary treatments

Commercial sources of dietary fats from the Midwest of the United States were obtained and analyzed for fatty acid profile prior to diet preparation (Table 1). In Exp. 1, 5 dietary treatments for each phase were formulated (Table 2): a control diet contained corn, soybean meal, and no addition of dietary fats and 4 additional diets by adding 3% SBO, 6% SBO, 3% CWG, or 6% CWG in each phase, respectively. In Exp. 2, 6 dietary treatments for each phase were formulated (Table 3): the control diet that was same as that in Exp. 1 and 5 additional diets by adding 6% SBO, 6% CWG, 6% palm oil, 6% animal-vegetable blend (AVB), or 6% tallow in each phase, respectively. The experimental diets used in each phase were formulated to meet or exceed all nutrient requirements of finishing pigs according to the Nutrient Requirements of Swine [11] and to have equivalent standardized ileal digestible lysine per Mcal of metabolizable energy. No antibiotic growth promoters were used and all diets were provided in a meal form.

**Table 1:** Analyzed fatty acid profile of dietary lipids

Item	Dietary lipids^a^				
	SBO	CWG	Palm oil	AVB	Tallow
Ether extract, % as-is	99.86	99.25	99.98	99.99	98.99
Fatty acids, % of ether extract					
Myristic (C14:0)	0.06	1.55	0.98	0.69	2.68
Myristoleic (C14:1)	–	0.13	–	0.08	0.51
Pentadecylic (C15:0)	0.01	0.13	0.04	0.06	0.44
Palmitic (C16:0)	10.63	23.36	43.07	13.60	22.65
Palmitoleic (C16:1)	0.09	2.52	0.16	0.90	2.66
Margaric (C17:0)	0.10	0.55	0.10	0.22	1.32
Heptadecenoic (C17:1)	0.06	0.35	0.02	0.14	0.61
Stearic (C18:0)	4.29	13.14	4.42	6.89	20.75
Elaidic (C18:1 *t*9)	0.04	1.22	0.13	1.62	5.26
Oleic (C18:1*n*9)	20.43	36.10	39.21	30.06	33.28
Vaccenic (C18:1*n*7)	2.32	4.80	0.00	3.49	2.66
Linoleic (C18:2)	52.77	11.97	10.26	34.64	2.58
Linolenic (C18:3)	7.66	0.57	0.15	3.74	0.17
Stearidonic (C18:4)	0.03	0.10	0.01	0.10	0.23
Arachidic (C20:0)	0.31	0.20	0.34	0.34	0.14
Eicosenoic (C20:1)	–	0.76	0.12	0.50	0.21
Behenic (C22:0)	0.35	0.02	0.05	0.27	0.00
Lignoceric (C24:0)	0.11	0.05	0.07	0.18	0.00
Saturated fatty acids	15.86	39.00	49.07	22.25	47.98
Monounsaturated fatty acids	22.94	45.88	39.64	36.79	45.19
Polyunsaturated fatty acids	60.46	12.64	10.42	38.48	2.98

^a^*SBO* Soybean oil, *CWG* Choice white grease, *AVB* Animal-vegetable blend

**Table 2:** Composition of experimental diets for Exp. 1

Item	Phase I^a^					Phase II^a^				
	Control	3% SBO	6% SBO	3% CWG	6% CWG	Control	3% SBO	6% SBO	3% CWG	6% CWG
Ingredients, %										
Ground corn	81.975	76.865	71.910	76.865	71.910	85.045	80.595	76.135	80.595	76.135
Soybean meal	14.845	16.985	18.955	16.985	18.955	12.000	13.450	14.900	13.450	14.900
SBO	–	3.000	6.000	–	–	–	3.000	6.000	–	–
CWG	–	–	–	3.000	6.000	–	–	–	3.000	6.000
Limestone	0.755	0.745	0.730	0.745	0.730	0.745	0.740	0.735	0.740	0.735
Dicalcium phosphate	1.220	1.215	1.210	1.215	1.210	1.130	1.125	1.125	1.125	1.125
Lysine HCl	0.365	0.345	0.335	0.345	0.335	0.325	0.325	0.325	0.325	0.325
*DL*-Methionine	0.025	0.030	0.040	0.030	0.040	0.010	0.015	0.020	0.015	0.020
*L*-Threonine	0.115	0.115	0.120	0.115	0.120	0.095	0.100	0.110	0.100	0.110
Salt	0.400	0.400	0.400	0.400	0.400	0.350	0.350	0.350	0.350	0.350
Vit-Min-mix^b^	0.300	0.300	0.300	0.300	0.300	0.300	0.300	0.300	0.300	0.300
Total	100.00	100.00	100.00	100.00	100.00	100.00	100.00	100.00	100.00	100.00
Energy and nutrients^c^										
Dry matter, %	90.39	90.63	88.59	90.11	88.68	89.22	85.88	87.38	89.22	87.12
Gross energy, Mcal/kg	3.77	3.97	4.16	3.97	4.05	3.76	3.92	4.10	3.96	4.08
Metabolizable energy, Mcal/kg	3.32	3.46	3.60	3.46	3.60	3.33	3.47	3.61	3.47	3.61
Crude protein, %	14.04	13.75	15.37	14.75	14.44	12.30	12.88	13.39	12.57	13.05
SID Lys, %	0.86	0.89	0.93	0.89	0.93	0.76	0.79	0.82	0.79	0.82
Ether extract, %	2.19	4.92	8.00	5.17	8.32	2.33	4.84	7.20	5.37	8.02
Ca, %	0.62	0.69	0.73	0.79	0.67	0.69	0.71	0.68	0.73	0.73
P, %	0.53	0.52	0.54	0.54	0.51	0.51	0.52	0.53	0.53	0.52
SID Lys^d^:Metabolizable energy, g/Mcal	2.59	2.57	2.58	2.57	2.58	2.28	2.28	2.27	2.28	2.27

^a^*SBO* Soybean oil, *CWG* Choice white grease

^b^Vitamin premix provided the following quantities of vitamins per kilogram of complete diet: 6,608 IU of vitamin A as retinyl acetate; 680 IU of vitamin D as cholecalciferol; *DL*-α-tocopheryl acetate, 88 mg; menadione sodium bisulfite complex 4 mg; riboflavin, 9 mg; vitamin B_12_, 35 μg; *D*-Ca-pantothenic acid, 24 mg; niacin, 33 mg; and choline chloride, 324 mg. Mineral premix provided the following quantities of mineral per kilogram of complete diet: Fe, 90 mg (FeSO_4_·H_2_O); Zn 100 mg (ZnO); Mn 20 mg (MnO); Cu 8 mg (CuSO_4_·H_2_O); I, 0.35 mg (CaI_2_); Se, 0.3 mg (Na_2_SeO_3_); and NaCl, 3 g

^c^Values for metabolizable energy and SID Lys were calculated from NRC (1998); all other nutrients were analyzed

^d^*SID Lys* Standardized ileal digestible lysine

**Table 3:** Composition of experimental diets for Exp. 2

Item	Phase I^a^						Phase II^a^					
	Control	6% SBO	6% CWG	6% Palm oil	6% AVB	6% Tallow	Control	6% SBO	6% CWG	6% Palm oil	6% AVB	6% Tallow
Ingredients, %												
Ground corn	81.975	71.910	71.910	71.910	71.910	71.910	85.045	76.135	76.135	76.135	76.135	76.135
Soybean meal	14.845	18.955	18.955	18.955	18.955	18.955	12.000	14.900	14.900	14.900	14.900	14.900
SBO	–	6.000	–	–	–	–	–	6.000	–	–	–	–
PO	–	–	–	6.000	–	–	–	–	–	6.000	–	–
AVB	–	–	–	–	6.000	–	–	–	–	–	6.000	–
CWG	–	–	6.000	–	–	–	–	–	6.000	–	–	–
Tallow	–	–	–	–	–	6.000	–	–	–	–	–	6.000
Limestone	0.755	0.730	0.730	0.730	0.730	0.730	0.745	0.735	0.735	0.735	0.735	0.735
Dicalcium phosphate	1.220	1.210	1.210	1.210	1.210	1.210	1.130	1.125	1.125	1.125	1.125	1.125
Lysine HCl	0.365	0.335	0.335	0.335	0.335	0.335	0.325	0.325	0.325	0.325	0.325	0.325
*DL*-Methionine	0.025	0.040	0.040	0.040	0.040	0.040	0.010	0.020	0.020	0.020	0.020	0.020
*L*-Threonine	0.115	0.120	0.120	0.120	0.120	0.120	0.095	0.110	0.110	0.110	0.110	0.110
Salt	0.400	0.400	0.400	0.400	0.400	0.400	0.350	0.350	0.350	0.350	0.350	0.350
Vit-Min-mix^b^	0.300	0.300	0.300	0.300	0.300	0.300	0.300	0.300	0.300	0.300	0.300	0.300
Total	100.00	100.00	100.00	100.00	100.00	100.00	100.00	100.00	100.00	100.00	100.00	100.00
Energy and nutrients^c^												
Dry matter, %	90.39	88.59	88.68	90.23	90.65	89.55	85.88	89.22	89.81	89.81	89.15	88.36
Gross energy, Mcal/kg	3.77	4.16	4.05	4.15	4.12	4.13	3.76	4.10	4.08	4.08	4.10	4.07
Metabolizable energy, Mcal/kg	3.32	3.60	3.60	3.60	3.60	3.60	3.33	3.60	3.60	3.60	3.60	3.60
Crude protein, %	14.04	15.37	14.44	15.13	15.21	15.37	12.30	13.39	13.05	13.08	13.75	13.64
SID Lys, %	0.86	0.93	0.93	0.93	0.93	0.93	0.76	0.82	0.82	0.82	0.82	0.82
Ether extract, %	2.19	8.00	8.32	8.17	7.94	7.84	2.33	7.20	8.02	7.91	8.18	7.20
Ca, %	0.62	0.73	0.67	0.73	0.61	0.65	0.69	0.68	0.73	0.75	0.61	0.73
P, %	0.53	0.54	0.51	0.53	0.50	0.53	0.51	0.53	0.52	0.50	0.50	0.52
SID Lys^d^:Metabolizable energy, g/Mcal	2.59	2.58	2.58	2.58	2.58	2.58	2.28	2.28	2.28	2.28	2.28	2.28

^a^*SBO* Soybean oil, *CWG* Choice white grease, *AVB* Animal-vegetable blend

^b^Vitamin premix provided the following quantities of vitamins per kilogram of complete diet: 6,608 IU of vitamin A as retinyl acetate; 680 IU of vitamin D as cholecalciferol; *DL*-α-tocopheryl acetate, 88 mg; menadione sodium bisulfite complex 4 mg; riboflavin, 9 mg; vitamin B_12_, 35 μg; *D*-Ca-pantothenic acid, 24 mg; niacin, 33 mg; and choline chloride, 324 mg. Mineral premix provided the following quantities of mineral per kilogram of complete diet: Fe, 90 mg (FeSO_4_·H_2_O); Zn 100 mg (ZnO); Mn 20 mg (MnO); Cu 8 mg (CuSO_4_·H_2_O); I, 0.35 mg (CaI_2_); Se, 0.3 mg (Na_2_SeO_3_); and NaCl, 3 g

^c^Values for metabolizable energy and SID Lys were calculated from NRC (1998); all other nutrients were analyzed

^d^*SID Lys* Standardized ileal digestible lysine

### Data collection

Pigs were weighed at the beginning of the trial and at the end of each phase, as well as feed consumption was recorded to determine average daily gain (ADG), average daily feed intake (ADFI), and gain:feed ratio (G:F). Pigs were scanned at the beginning and the end of both experiments using an Aloka Model SSD-500 scanner fitted with a VST-5021-3 probe (Corometrics Medical Systems, Wallingford, CT). This equipment was a convex sector/linear scanner. The probe had a frequency of 3 MHz, a scanning width of 125 mm, and a diagnostic depth of up to 283 mm. Each individual pig was restrained in a crate during scanning and its live weight was recorded. A longitudinal scan was taken parallel to the long axis of the pig immediately anterior to the last rib, 6.5 cm from the midline on the left side. Peanut oil was used for all scans to achieve contact between probe and body surface of the pig. Measurements taken on the longitudinal scan were backfat depth at the last rib and 10^th^ rib and longissimus muscle depths at the last rib and 10^th^ rib. All procedures were based on the methods described in Cisneros et al. [12].

### Chemical analysis

All analyses for the diets were performed in duplicate samples and repeated if results from duplicate diet samples varied more than 5% from the mean. All analysis were completed prior to animal trials. The dry matter of diets was determined by oven drying at 135 °C for 2 h [13]. The gross energy of diets was measured using an adiabatic bomb calorimeter (Model 6300, Parr Instruments, Moline, IL). The concentration of N in diets was measured using the combustion method [13] on an Elementar Rapid N-cube protein/nitrogen apparatus (Elementar Americas Inc., Mt. Laurel, NJ). The concentration of crude protein was calculated as N × 6.25. The concentration of crude fat in diets was measured using the petroleum ether extraction method [13] on a Soxtex 2050 automated analyzer (FOSS North America, Eden Prairie, MN). The concentration of calcium in diets was measured using inductively coupled plasma atomic emission spectroscopy in the Experiment Station Chemical Labs in University of Missouri. The phosphorus contents in diets were determined using gravimetric method [13].

### Statistical analysis

Normality of data was confirmed and outliers were tested using the UNIVARIATE procedure of SAS (SAS Institute Inc., Cary, NC). Data were analyzed by ANOVA using the MIXED procedure with pen as the experimental unit. The statistical model included diet as a fixed effect and replicate as a random effect. Least squares means were calculated using the Least-Significant Difference test and means were separated using the PDIFF statement and adjusted with Tukey. Orthogonal contrasts were used to determine the differences between control versus fat, fat source, fat level, and the interaction of fat source and fat level in Exp. 1 and the difference between control versus fat in Exp. 2. For the analysis of backfat depth and muscle depth, pig body weight was used as a covariate. An alpha level of 0.05 was used to assess significance among means. If the *P*-value was between 0.05 and 0.10, responses were viewed as tendencies.

## Results and discussion

### Fatty acid composition

The five dietary fats used in this study had widely different fatty acid compositions (Table 1). The ratio of unsaturated fatty acids to saturated fatty acids were 5.26 in SBO, 1.50 in CWG, 1.02 in palm oil, 3.38 in AVB, and 1.00 in tallow, respectively. The most saturated were tallow and palm oil, followed in order of increasing unsaturation by CWG, AVB, and SBO.

The composition of fatty acids in SBO, CWG, and tallow corresponds to published values [9, 14, 15]. However, the fatty acid composition of AVB used in Exp. 2 is more saturated than the published values [14]. Lipid digestibility and availability are influenced by physicochemical properties of dietary lipids, including the chain length and degree of unsaturation of fatty acids, the position of fatty acids on the glycerol, and the amount of moisture, insoluble impurities, and unsaponifiable materials. The digestibility of lipids increases as the degree of saturation and chain length decreases [16, 17].

### Growth performance

Supplemental dietary fats reduced (*P* < 0.05) ADFI in phase II (3.350 vs. 3.539 kg/d) and the overall period (3.251 vs. 3.415 kg/d) and increased (*P* < 0.01) G:F in phase II (0.332 vs. 0.300) and the overall period (0.365 vs. 0.333) compared with the control diet (Table 4). No differences were observed in ADG or BW of pigs fed fats compared with those fed the control diet. The higher level of 6% fat tended (*P* < 0.10) to decrease ADFI and increased (*P* < 0.05) G:F in phase II and the overall period compared with the lower level of 3% fat. There were interactions observed in ADG (*P* < 0.10) and G:F (*P* < 0.05) in the early stage of this experiment, as increasing CWG from 3% to 6% increased ADG and G:F, but the direction was opposite with SBO.

**Table 4:** Effects of dietary soybean oil (SBO) and choice white grease (CWG) on growth performance of finishing pigs, Exp. 1^a^

Item	Dietary treatment					SEM	*P*-value			
	Control	3% SBO	6% SBO	3% CWG	6% CWG		Control vs. Fat^b^	Source^c^	Level^d^	Source×Level^e^
Initial BW, kg	65.89	63.94	65.88	64.58	63.61	0.852	0.15	0.35	0.57	0.10
D 21 BW, kg	90.66	89.58	89.90	90.88	91.10	1.196	0.83	0.30	0.82	0.97
Final BW, kg	120.82	120.85	122.59	120.17	123.84	1.678	0.58	0.87	0.12	0.57
Phase I (d 0 to 21)										
ADG,^f^ kg	1.180	1.229	1.144	1.252	1.309	0.036	0.18	< 0.05	0.70	0.054
ADFI,^f^ kg	3.168	3.100	2.936	3.152	3.022	0.089	0.26	0.44	0.11	0.85
G:F^f^	0.382	0.406	0.395	0.403	0.443	0.009	< 0.01	< 0.05	0.12	< 0.05
Phase II (d 22 to 49)										
ADG,^f^ kg	0.908	1.117	1.167	1.046	0.845	0.119	0.31	0.11	0.53	0.30
ADFI,^f^ kg	3.539	3.458	3.299	3.379	3.264	0.082	< 0.05	0.50	0.10	0.79
G:F^f^	0.300	0.324	0.354	0.310	0.339	0.010	< 0.01	0.13	<.01	0.94
Overall										
ADG,^f^ kg	1.032	1.088	1.157	1.134	1.058	0.077	0.38	0.74	0.97	0.35
ADFI,^f^ kg	3.415	3.339	3.178	3.304	3.183	0.075	< 0.05	0.84	0.07	0.79
G:F^f^	0.333	0.357	0.371	0.348	0.383	0.007	< 0.01	0.84	< 0.01	0.15

^a^Data were least squares means of 9 observations per treatment

^b^Contrast analyses between control diet and the average of other 4 diets

^c^Contrast analyses between the average of two SBO diets and the average of two CWG diets

^d^Contrast analyses between the average of two 3% fat diets and the average of two 6% fat diets

^e^Contrast analyses between the average of 3% SBO + 6% CWG and the average of 3% CWG + 6% SBO

^f^*ADFI* Average daily feed intake, *ADG* Average daily gain, *G:F* Gain:feed

Pigs fed diets supplemented with 6% dietary fats had greater (*P* < 0.01) ADG (1.293 vs. 1.132 kg/d) in phase I, lower (*P* < 0.01) ADFI in phase II (3.507 vs. 3.877 kg/d) and the overall period (3.395 vs. 3.654 kg/d; Table 5). Pigs fed diets supplemented with 6% dietary fats also had greater (*P* < 0.01) G:F in phase I (0.411 vs. 0.357), phase II (0.332 vs. 0.302), and the overall period (0.362 vs. 0.322) than pigs fed the control diet.

**Table 5:** Effects of different dietary fats on growth performance of finishing pigs, Exp. 2^d^

Item	Dietary treatment^e^						SEM	*P*-value	
	Control	6% SBO	6% CWG	6% Palm oil	6% AVB	6% Tallow		Diet	Control vs. Fat^f^
Initial BW, kg	73.26	74.11	72.80	73.48	73.28	71.48	0.711	0.18	0.72
D 19 BW, kg	94.77	97.49	98.48	98.74	96.89	96.41	1.212	0.23	< 0.05
Final BW, kg	127.53	129.12	130.40	132.63	130.03	128.90	1.721	0.43	0.16
Phase I (d 0 to 19)									
ADG,^g^ kg	1.132^c^	1.230^bc^	1.352^a^	1.329^ab^	1.243^b^	1.312^ab^	0.038	< 0.05	< 0.01
ADFI,^g^ kg	3.208	3.107	3.150	3.298	3.092	3.211	0.066	0.67	0.52
G:F^g^	0.357^b^	0.399^a^	0.428^a^	0.406^a^	0.411^a^	0.411^a^	0.012	< 0.01	< 0.01
Phase II (d 20 to 47)									
ADG,^g^ kg	1.170	1.130	1.140	1.210	1.184	1.160	0.041	0.91	0.69
ADFI,^g^ kg	3.877^a^	3.353^c^	3.457^c^	3.690^ab^	3.539^bc^	3.494^bc^	0.079	< 0.01	< 0.01
G:F^g^	0.302^b^	0.336^a^	0.329^a^	0.328^a^	0.334^a^	0.332^a^	0.007	< 0.01	< 0.01
Overall									
ADG,^g^ kg	1.155	1.170	1.226	1.259	1.208	1.222	0.032	0.87	0.53
ADFI,^g^ kg	3.654^a^	3.271^c^	3.355^c^	3.559^ab^	3.390^bc^	3.399^bc^	0.067	< 0.05	< 0.01
G:F^g^	0.322^b^	0.360^a^	0.367^a^	0.357^a^	0.362^a^	0.362^a^	0.007	< 0.01	< 0.01

^a,b,c^Within a row, means without a common superscript differ (*P* < 0.05)

^d^Data are least squares means of 8 observations per treatment

^e^*SBO* Soybean oil, *CWG* Choice white grease, *AVB* Animal-vegetable blend

^f^*P*-value for control vs. fat was based on contrast analyses between control diet and the average of other 5 diets added with fat

^g^*ADFI* Average daily feed intake, *ADG* Average daily gain, *G:F* Gain:feed

The improved feed efficiency observed in late finishing pigs in both experiments as the dietary energy density was increased by addition of fats was expected and agrees with previously published research [7, 9, 18–20]. The observation of reduced ADFI as a result of increasing the level of dietary fats also agrees with previous observations [9, 19], because pigs often reduce feed intake as the dietary energy concentration increases [1, 2]. Supplemental fat has previously increased growth rate in some conditions but not in others, as reviewed by Pettigrew and Moser [1]. In the present case it increased growth rate during phase I only of one of the two experiments.

In many cases, increasing energy intake by pigs results in increasing protein accretion. Often, especially in older pigs, the protein accretion rate reaches a maximum constrained by other factors, so further increases in energy intake do not increase protein accretion. Young pigs often fail to consume enough energy to reach the maximum protein accretion rate [21]. Therefore, protein accretion rate and the associated growth rate are more sensitive to energy status in younger animals than in older ones. This is likely the reason dietary effects on growth rate were found in phase I but not in phase II.

The faster growth during the sensitive early period on diets containing CWG than on those containing SBO in both of the present experiments. The observations support the greater net energy value of CWG than of SBO for growing pigs but not for finishing pigs previously reported by Kil et al. [7]. It also confirms a practical benefit of that greater net energy value. The accompanying superiority of CWG over AVB in the present data suggests this phenomenon may be a general response to degree of unsaturation; the concentrations of unsaturated fatty acids in SBO (83%) and AVB (75%) are much higher than in CWG (58%) used in the present experiment (Table 1).

The superiority of CWG over SBO for younger animals shown here occurs in spite of the higher digestibility of unsaturated lipids frequently reported [22], suggesting the absorbed lipids may be used more efficiently in CWG than SBO. This hypothesis was partially supported by the greater lipid deposition in growing pigs fed CWG compared with pigs fed SBO [7]. Potential mechanisms for greater efficiency include reduction in oxidative stress, reduction in turnover of triacylglycerols, and less fatty acid oxidation [7]. A reduction in fatty acid oxidation would be especially important because the predicted energetic efficiency of digested dietary lipids is 66% for ATP production, while the efficiency is 90% if the digested dietary lipids are directly incorporated into body lipids [23].

It may be inappropriate to extrapolate the greater energy value of CWG found here with finishing pigs to young recently-weaned pigs. Digestibility of saturated fats is sharply lower than of polyunsaturated ones in pigs immediately after weaning [3].

### Carcass characteristics

In Exp. 1, supplementation of dietary fat did not affect backfat depth or muscle depth of finishing pigs, with the exception that the addition of fat tended (*P* = 0.057) to increase final backfat depth at the last rib (2.329 vs. 2.137 cm) compared with the control diet (Table 6). Pigs fed CWG had a bigger (*P* < 0.05) increase in muscle depth at the 10^th^ rib (1.439 vs. 1.237 cm) than pigs fed SBO. In Exp. 2, supplementation of 6% fats did not affect backfat depth or muscle depth of finishing pigs (Table 7). Among fat sources, pigs fed with 6% palm oil had thicker (*P* < 0.05) backfat at the 10th rib than pigs fed with 6% SBO, AVB, and tallow, while pigs fed 6% AVB or tallow had greater (*P* < 0.05) muscle depth at the last rib than pigs fed with 6% CWG and palm oil.

**Table 6:** Effects of dietary soybean oil (SBO) and choice white grease (CWG) on backfat depth at last rib, backfat depth at 10^th^ rib, muscle depth at last rib, and muscle depth at 10^th^ rib of finishing pigs, Exp. 1^a^

Item	Dietary treatment					SEM	*P*-value			
	Control	3% SBO	6% SBO	3% CWG	6% CWG		Control vs. Fat^b^	Source^c^	Level^d^	Source×Level^e^
Initial, cm										
Backfat depth at last rib	1.223	1.284	1.281	1.320	1.279	0.042	0.17	0.69	0.61	0.66
Backfat depth at 10^th^ rib	1.239	1.238	1.277	1.263	1.243	0.036	0.69	0.91	0.78	0.43
Muscle depth at last rib	3.420	3.405	3.414	3.263	3.663	0.094	0.88	0.57	< 0.05	< 0.05
Muscle depth at 10^th^ rib	3.517	3.513	3.532	3.322	3.595	0.076	0.76	0.41	0.062	0.12
Final, cm										
Backfat depth at last rib	2.137	2.343	2.338	2.266	2.370	0.087	0.057	0.79	0.58	0.54
Backfat depth at 10^th^ rib	2.208	2.435	2.359	2.338	2.348	0.090	0.11	0.55	0.72	0.64
Muscle depth at last rib	4.971	4.802	4.781	4.722	4.972	0.104	0.20	0.60	0.29	0.20
Muscle depth at 10^th^ rib	4.982	4.785	4.730	4.781	4.945	0.111	0.18	0.35	0.64	0.33
Difference, cm										
Backfat depth at last rib	0.889	1.053	1.048	0.941	1.081	0.080	0.13	0.63	0.41	0.39
Backfat depth at 10^th^ rib	0.961	1.170	1.083	1.074	1.087	0.083	0.15	0.58	0.67	0.56
Muscle depth at last rib	1.462	1.462	1.296	1.458	1.420	0.113	0.68	0.59	0.37	0.59
Muscle depth at 10^th^ rib	1.414	1.315	1.158	1.455	1.423	0.087	0.45	< 0.05	0.28	0.49

^a^Data were least squares means of 9 observations per treatment

^b^Contrast analyses between control diet and the average of other 4 diets

^c^Contrast analyses between the average of two SBO diets and the average of two CWG diets

^d^Contrast analyses between the average of two 3% fat diets and the average of two 6% fat diets

^e^Contrast analyses between the average of 3% SBO + 6% CWG and the average of 3% CWG + 6% SBO

**Table 7:** Effects of different dietary fats on backfat depth at last rib, backfat depth at 10^th^ rib, muscle depth at last rib, and muscle depth at 10^th^ rib of finishing pigs, Exp. 2^d^

Item	Dietary treatment^e^						SEM	*P*-value	
	Control	6% SBO	6% CWG	6% Palm oil	6% AVB	6% Tallow		Diet	Control vs. Fat^f^
Initial, cm									
Backfat depth at last rib	1.430	1.378	1.379	1.496	1.436	1.334	0.052	0.19	0.66
Backfat depth at 10^th^ rib	1.341	1.445	1.333	1.449	1.349	1.306	0.047	0.11	0.49
Muscle depth at last rib	4.015^a^	3.868^ab^	3.878^ab^	3.694^b^	4.003^ab^	3.949^ab^	0.088	0.09	0.16
Muscle depth at 10^th^ rib	4.009^a^	3.925^abc^	3.793^bc^	3.734^c^	4.075^a^	3.980^ab^	0.083	< 0.05	0.24
Final, cm									
Backfat depth at last rib	2.232	2.193	2.236	2.473	2.224	2.168	0.099	0.27	0.81
Backfat depth at 10^th^ rib	2.261^b^	2.302^b^	2.383^ab^	2.624^a^	2.211^b^	2.282^b^	0.105	< 0.05	0.40
Muscle depth at last rib	4.933^ab^	4.947^ab^	4.831^b^	4.822^b^	5.172^a^	5.123^a^	0.103	0.07	0.69
Muscle depth at 10^th^ rib	4.878	4.906	4.873	4.770	5.138	5.068	0.111	0.15	0.56
Difference, cm									
Backfat depth at last rib	0.819	0.818	0.849	0.965	0.782	0.846	0.082	0.80	0.73
Backfat depth at 10^th^ rib	0.948	0.861	1.039	1.137	0.857	0.995	0.091	0.12	0.78
Muscle depth at last rib	0.945	1.137	0.940	1.129	1.177	1.101	0.093	0.44	0.19
Muscle depth at 10^th^ rib	0.895	1.014	1.068	1.063	1.066	1.062	0.109	0.91	0.24

^a,b,c^Within a row, means without a common superscript differ (*P* < 0.05)

^d^Data are least squares means of 8 observations per treatment

^e^*SBO* Soybean oil, *CWG* Choice white grease, *AVB* Animal-vegetable blend

^f^*P*-value for control vs. fat was based on contrast analyses between control diet and the average of other 5 diets added with fat

Results of Exp. 1 indicate that the inclusion of dietary fats may increase backfat depth, but this was not the case in Exp. 2. Only the diet containing 6% palm oil increased backfat depth compared with the control diet and other diets containing different fat sources. The inconsistency agrees with reports in the literature, as most research [1, 2, 19, 24] found that feeding various fat sources reduces leanness, but some [9, 19] found no effects.

## Conclusions

In conclusion, the greater energy provided by CWG than SBO at the early stage of both experiments supports the reported observations from Kil et al. [7]. These observations indicate that the relatively saturated CWG has a greater net energy value than SBO containing greater amounts of unsaturated fatty acids when they are included in the diet for growing pigs. Results from both experiments indicate that dietary fat added as 6% of the diet improves feed efficiency of finishing pigs but may reduce carcass leanness. Different fats produced different practical results that may be consistent with their different energy values.

## Abbreviations

ADFI: Average daily feed intake
ADG: Average daily gain
AVB: Animal-vegetable blend
CWG: Choice white grease
G:F: Gain:Feed
SBO: Soybean oil
